# Is the recurrence rate of incisional hernia is affected by mesh positioning?

**DOI:** 10.1016/j.amsu.2021.01.002

**Published:** 2021-01-09

**Authors:** Rashid Ibrahim, Talal Alshehri, Sabry Abounozha

**Affiliations:** a University Hospitals Plymouth NHS Trust, Plymouth, UK; b Imam Abdulrahman Alfaisal Hospital, Riyadh, Saudi Arabia; c Northumbria Healthcare NHS Foundation Trust, Northumbria, UK

**Keywords:** Incisional hernia, Onlay, Recurrence, Sublay

## Abstract

A best evidence topic has been constructed using a described protocol. The three-part question addressed was: In open mesh repair of incisional hernia, which technique has a lower recurrence rate, Sublay or Onlay? The best evidence showed that there is no statistically significant difference in the rate of recurrence among the two techniques.

## Highlights

• Comparison of recurrence rate among patients who underwent Onlay vs Sublay mesh repair of incisional hernia.
• The best evidence showed that no statistical difference in the recurrence rate between Sublay and Onlay mesh of incisional hernia.

## 1 Introduction

This BET was designed using a framework outlined by the International Journal of Surgery [1]. This format was used because a preliminary literature search suggested that the available evidence is of insufficient quality to perform a meaningful meta-analysis. A BET provides evidence-based answers to common clinical questions, using a systematic approach of reviewing the literature.

## 2 Clinical scenario

You are consenting a 40 year old morbidly obese male with recurrent incisional hernia, for open mesh repair. The previous surgery was suture repair; you are wondering which technique offers lower recurrence rate Onlay or Sublay?

## 3 Three-part question

[In open mesh repair of incisional hernia] [which techniques has lower recurrence rate] [Sublay or Onlay]?

## 4 Search strategy

A. Embase 1974 to October 2020 using the OVID interface:

[Incisional hernia] AND [mesh ] AND [repair OR repairs ] AND [onlay] AND [sublay]AND[surgical site infection OR SSI OR wound infection OR infection].

B. Medline using the PubMed interface:

[Incisional hernia] AND [mesh ] AND [repair OR repairs] AND [onlay] AND [sublay]AND[recurrence ].

The results were limited to English articles and human studies.

## 5 Search outcome

A total of 65 articles were identified after the removal of duplicates. Of these 50 were excluded on the basis of title and abstract. After full-text assessment of 15 articles another 8 articles were excluded because they did not include the information needed to compare the two techniques. A total of 7 articles (4 randomized controlled trials, one prospective and 2 retrospective studies) were identified to provide the best evidence to answer the question.

## 6 Result: see the table

**Table:** 

Author, date of publication, journal and country	Study type and level of evidence	Patient group Follow up	Outcomes	Key results		Additional comments
Demetrashvili et al. 2017 International Journal of Surgery Georgia.	Randomized controlled trial level II	180 patients Group 1: 90 onlay Group2: 90 sublay Follow-up for the Sublay = (4.3 ± 1.2 years), Onlay = (4.6 ± 1.0 years)	Primary endpoint: recurrence rate	Group1 = 4(5.1%) Group2 = 2(2.6%) P = 0.68 Difference is not statistically significant		-Single centre, -large sample size, -Short period of follow up
Sevinç, et al. 2018 Turk J Surg Turkey	Randomized controlled trial level II	Total of 100 with incisional hernia **Group 1**: 50 sublay **Group2**: 50 onlay median follow-up was 37.1 (26.6–46.5) months	Primary endpoint: recurrence rate	Group1 = 3(6%) Group2 = 1 (2%) p = 0.307 Difference is not statistically significant		-Single centre, -Small sample size, -Short period of follow up
Venclauskas et al. 2010 Hernia Lithuania	Randomized controlled trial level II	Total 107 patients underwent mesh repair Group1: 57onlay Group2: 50 sublay median follow-up 12 months	Primary endpoint: recurrence rate	Group1: 6 (10.5%) Group2: 1 (2%) (P = 0.077). Difference is not statistically significant		Single centre, -Small sample size, -Short period of follow up -Nothing mentioned about the methods of diagnosis of SSI
Manzoor et al. 2019 J Coll Physicians Surg Pak Pakistan	Multicenter, Randomized, Controlled Trial levelII	Total 65 patients underwent mesh repair Group1: 33onlay Group2: 32 sublay median follow-up Six months.	Primary endpoint: recurrence rate	Group1: 0 (0.0%) Group2: 0(0.0%) (P = 1). Difference is not statistically significant		-Small sample size, -Short period of follow up
Kumar et al. 2012 Indian J Surg India	Prospective study level III	Total 63 patients Randomized into: **Group 1**: 45 onlay **Group 2**: 18 sublay follow-up 5 years	Primary endpoint: recurrence rate	**Group 1** = 10.8% **Group 2=** 9% Difference is not statistically significant	-Single centre, -Small sample size -No randomization - sample size is not equal between 2 groups	
John J. Gleysteen, Arch Surg. 2009 UK	Retrospective study level III	A total of 125 patients **Group 1: 75 onlay Group 2: 50 sublay** Follow-up periods averaged 64 months	Primary endpoint: recurrence rate	**Group1** = 15 (20.0%) **Group2** = 2 (4.0%) (P = 0.02) Difference is statistically significant	-Single centre, -small sample size, -Retrospective	
Reilingh et al. Hernia (2004) Netherlands	Retrospective cohort study, level III	80 patients underwent Mesh repair of incisional hernia Group 1: 13 onlay Group 2: 23 sublay	Primary endpoint: recurrence rate	**Group 1** = 3(23%) **Group 2** = 10(43%) (P = 0.08) Statically significant	-Single centre, -small sample size, -Retrospective	

## 7 Discussion

Several clinical trials proved that the mesh repair of incisional hernia has lower recurrence rate compared with suture repair [2].The risk factors for recurrence of incisional hernia have been classified into patient related (e.g., older age, obesity, diabetes, smoking, immunosuppression), hernia related (e.g., size and location) [3], and surgically related (e.g., experience, skills) [4]. It is still debatable whether the position of the mesh during the repair is associated with increased risk of recurrence. The aim from this review is to evaluate the rate of recurrence of incisional hernia among patients who underwent onlay versus sublay open mesh repair.

Reilingh et al., in 2005 [5] published retrospective study in order to assess the recurrence rate among onlay and sublay techniques, their conclusion was; sublay mesh repair has higher incidence of recurrence compared to onlay mesh. In contrast to this finding; Gleysteen in 2009 [6], conducted another retrospective study which showed that the incidence of recurrence is actually higher among patients who underwent onlay rather than sublay technique.

However, despite these contradicting results,we have also reviewed another 5 studies including 4 randomized controlled trials and one prospective trial that showed no statistically significant difference in the recurrence rate of incisional hernia between Only and Sublay mesh repair these studies were conducted by Demetrashvili et al., Sevinç et al. Venclauskas, Manzoor et al., Kumar et al. and [[7], [8], [9], [10], [11]].

## 8 Clinical bottom line

The best evidence showed no statistically significant difference in the recurrence rate between only and Sublay mesh repair of incisional hernia.

## 9 Limitation of this review

1. Small sample size in most articles
2. Shorter period of follow in most articles.

## Author contribution

(RI): conducted the literature search and wrote the paper.

(TA): assisted in the literature search, editing of writing.

(SA): assisted in the literature search and Writing of paper.

## Provenance and peer review

Not commissioned, externally peer reviewed.

## Annals of medicine and surgery

The following information is required for submission. Please note that failure to respond to these questions/statements will mean your submission will be returned. If you have nothing to declare in any of these categories then this should be stated.

Please state any conflicts of interest.

All authors must disclose any financial and personal relationships with other people or organisations that could inappropriately influence (bias) their work. Examples of potential conflicts of interest include employment, consultancies, stock ownership, honoraria, paid expert testimony, patent applications/registrations, and grants or other funding.

Please state any sources of funding for your research.

All sources of funding should be declared as an acknowledgement at the end of the text. Authors should declare the role of study sponsors, if any, in the collection, analysis and interpretation of data; in the writing of the manuscript; and in the decision to submit the manuscript for publication. If the study sponsors had no such involvement, the authors should so state.

## Ethical approval

Research studies involving patients require ethical approval. Please state whether approval has been given, name the relevant ethics committee and the state the reference number for their judgement.

## Consent

Studies on patients or volunteers require ethics committee approval and fully informed written consent which should be documented in the paper.

Authors must obtain written and signed consent to publish a case report from the patient (or, where applicable, the patient's guardian or next of kin) prior to submission. We ask Authors to confirm as part of the submission process that such consent has been obtained, and the manuscript must include a statement to this effect in a consent section at the end of the manuscript, as follows: “Written informed consent was obtained from the patient for publication of this case report and accompanying images. A copy of the written consent is available for review by the Editor-in-Chief of this journal on request”.

Patients have a right to privacy. Patients’ and volunteers' names, initials, or hospital numbers should not be used. Images of patients or volunteers should not be used unless the information is essential for scientific purposes and explicit permission has been given as part of the consent. If such consent is made subject to any conditions,the Editor in Chief must be made aware of all such conditions.

Even where consent has been given, identifying details should be omitted if they are not essential. If identifying characteristics are altered to protect anonymity, such as in genetic pedigrees, authors should provide assurance that alterations do not distort scientific meaning and editors should so note.

## Registration of Research Studies

In accordance with the Declaration of Helsinki 2013, all research involving human participants has to be registered in a publicly accessible database. Please enter the name of the registry and the unique identifying number (UIN) of your study.

You can register any type of research at http://www.researchregistry.com to obtain your UIN if you have not already registered. This is mandatory for human studies only. Trials and certain observational research can also be registered elsewhere such as: ClinicalTrials.gov or ISRCTN or numerous other registries.

## Guarantor

The Guarantor is the one or more people who accept full responsibility for the work and/or the conduct of the study, had access to the data, and controlled the decision to publish.
